# Reduced expression of TAZ inhibits primary cilium formation in renal glomeruli

**DOI:** 10.1038/s12276-022-00730-2

**Published:** 2022-02-17

**Authors:** Jae Hee Jun, Eun Ji Lee, Minah Park, Je Yeong Ko, Jong Hoon Park

**Affiliations:** Department of Biological Science, Sookmyung Women’s University, Seoul, 04310 Republic of Korea

**Keywords:** Mechanisms of disease, Kidney diseases

## Abstract

Renal primary cilia are antenna-like organelles that maintain cellular homeostasis via multiple receptors clustered along their membranes. Recent studies have revealed that YAP/TAZ, key paralogous effectors of the Hippo pathway, are involved in ciliogenesis; however, their independent roles need to be further investigated. Here, we analyzed the renal phenotypes of kidney-specific TAZ knockout mice and observed ciliary defects only in glomeruli where mild cysts were formed. This finding prompted us to verify the role of TAZ specifically in renal tubule ciliary regulation. Therefore, we investigated the effects of TAZ silencing and compared them to those of YAP knockdown using three different types of renal tubular cells. We found that the absence of TAZ prevented proper cilia formation in glomerular cells, whereas it had a negligible effect in collecting duct and proximal tubule cells. IFT and NPHP protein levels were altered because of TAZ deficiency, accompanied by ciliary defects in glomerular cells, and ciliary recovery was identified by regulating some NPHP proteins. Although our study focused on TAZ, ciliogenesis, and other ciliary genes, the results suggest the very distinct roles of YAP and TAZ in kidneys, specifically in terms of ciliary regulation.

## Kidney cysts: Formation of signaling antennae

The roles of two regulatory proteins in the kidneys have been further clarified and provide insights into cilia defects and cyst formation. Cilia are organelles that act as ‘antennae’ for cell signaling in many tissues. Recent studies have highlighted two proteins involved in kidney cilia formation, YAP and TAZ, but little is known about their roles. Jong Hoon Park and co-workers at Sookmyung Women’s University in Seoul, South Korea, examined the role of TAZ in the regulation of kidney tubule cilia in mice. They explored the effects of silencing TAZ or YAP expression in different types of kidney tubule cells. TAZ deficiency but not YAP deficiency prevented correct cilia formation in the glomeruli, blood vessels that filter waste in the kidneys, and the resulting defects led to mild cyst generation.

## Introduction

Primary cilia are sensory organelles that act as antennae to transmit signals within and outside the cell. There are two major types of cilia, namely, motile and nonmotile cilia, both of which are alpha- and beta-tubulin microtubule-based organelles and are composed of an axoneme of 9 doublet microtubules^1^. The difference between the two is that motile cilia have a central pair of microtubules, a 9 + 2 structure and mobility, whereas nonmotile cilia have a 9 + 0 structure and lack a central pair of microtubules and dynein arms. Primary cilia are nonmotile cilia. To better understand the structure of primary cilia, we focused on the axoneme, transition zones, and basal body. The basal body composition of primary cilia was studied by assessing docking of the mother centriole of the centrosome on the cell plasma membrane in the G0 phase of the cell cycle. After a distal appendage of the basal body is anchored to the membrane, a ciliary pocket is formed to assemble the axoneme, which is used in transport of the intraflagellar complex. Kinesin-2 transports cargo proteins such as intraflagellar transport (IFT) complex B to the ciliary tip and, upon cell cycle reentry, the dynein-2 retrograde IFT complex A is transported from the ciliary tip to the base of the cilium, causing the primary cilium structure to disassemble. Thus, IFT proteins regulate ciliary assembly and transport proteins to maintain homeostasis^2^.

Primary cilia are organelles that exist in cells in many mammalian organs and tissues, including the kidneys, pancreas, sensory organs, and brain neuroepithelum. Ciliogenesis, involving approximately 1800 genes, has been reported to be associated with the Shh, Wnt, Notch, and mTOR signaling pathways, which regulate cellular homeostasis, proteostasis, and autophagy. As cell signaling is controlled through primary cilia in many organs, ciliary defects cause diseases referred to as ciliopathies. Genetic defects lead to ciliopathy phenotypes such as polydactyly, sensorineural deafness, renal fibrosis and inflammation, cystic renal disease, and retinal degeneration^3–5^.

Yes-associated protein (YAP) and transcriptional coactivator with PDZ-binding motif (TAZ; WWTR1) are homologous to each other and are known to be located downstream of the Hippo pathway. The Hippo pathway was first identified in *Drosophila* and contains tumor-suppressing genes that regulate organ size in^6,7^. The tissue overgrowth phenotype was consistent with the occurrence of mutations in the *hpo*, *sav*, and *wts* genes in *Drosophila*. Later, *Wts/Mats* was found to regulate the Yki protein, which is phosphorylated by 14-3-3 proteins to regulate its activation. The Hippo pathway observed in *Drosophila* is significantly conserved in mammals^8^. In mammalian signaling, SAV1 and MST1/2 activation leads to the activation of LATS1/2 and MOB1A/B. YAP/TAZ, the terminal downstream effectors of Hippo signaling, are phosphorylated to form a complex with 14-3-3 or degraded in the cytoplasm. Conversely, if the upstream proteins of the Hippo pathway are phosphorylated and inactivated, YAP/TAZ are activated and transported into the nucleus, where they act as transcriptional cofactors with TEAD^9^.

YAP and TAZ are human orthologs of *Drosophila* Yorkie, and they are paralogs of each other; thus, their structures are slightly different but include a TEAD-binding domain, PDZ-binding domain, and WW domain, among others^7^. YAP and TAZ, the major effectors of Hippo signaling, play a variety of roles in regulating cell contact inhibition, epithelial-mesenchymal transition, development, proliferation, and differentiation^7^. In tumors, they control cell growth or stiffness and are characterized as oncogenes^10,11^. Although YAP/TAZ are expected to have similar roles because of their similar structures, it has recently been proposed that they play individual and distinct roles. Some studies have suggested that they may play not only different but also conflicting roles^9,12–14^. Further research is needed to determine the precise relationship between YAP and TAZ.

The role of YAP/TAZ, the major effectors of Hippo signaling, in the regulation of renal cystogenesis and ciliogenesis has been investigated in various studies. Glomerulocystic and ciliary defect phenotypes have been observed in TAZ-deficient mice, and it has been reported that the interaction between Glis3 and TAZ modulates ciliogenesis^15,16^. YAP is known to be involved in ciliogenesis by modulating actin remodeling factors^17,18^. However, the independent functions of YAP/TAZ in primary cilia formation in renal tubules have not yet been elucidated. In this study, we investigated the mechanism by which TAZ deficiency in the kidney affects primary cilia phenotypes in each renal tubule type. Furthermore, we assessed the possibility of distinct functions of YAP and TAZ in cilia formation.

## Materials and methods

### Cell culture

mIMCD cells were grown in DMEM/F-12 medium (LM 002-08, Welgene, Gyeongsan-si, Gyeongsangbuk-do, Republic of Korea) supplemented with 10% fetal bovine serum (16000-044, Gibco) and 1% penicillin/streptomycin (LM002-04, Welgene). TKPTS cells were purchased from ATCC and grown in DMEM/F-12 medium (LM 002-08, Welgene) supplemented with 10% FBS, 0.06% 10 mg/mL insulin (Cat# 19278, Sigma, St. Louis, MO, 63178, USA), and 1% P/S. SV40MES13 cells were also purchased from ATCC and grown in a 3:1 mixture of DMEM (LM001-05, Welgene) and Ham’s F12 medium (LM 010-01, Welgene) supplemented with 14 mM HEPES (H3537, Sigma), 5% FBS, and 1% P/S. Primary ciliogenesis was induced under serum withdrawal in FBS-free medium for most cell lines. For the NIH3T3 cell line, medium containing 0.5% FBS was used. Usually, primary cilia were generated under serum starvation (no FBS) for 24–48 h.

### Transfection

Cells were transfected with siRNAs targeting Yap (sc-38638, Santa Cruz Biotechnology, Dallas, Texas, 75220, U.S.A.) or Taz (sc-38569, Santa Cruz Biotechnology) at a concentration of 10–30 nM for 24 h or 48 h using Lipofectamine RNAiMAX (13778150, Thermo Fisher Scientific), according to the manufacturer’s instructions, one day after seeding. Cells were transfected with siRNAs targeting Nphp6 (sc-72866, Santa Cruz Biotechnology) at a concentration of 30 nM and Nphp9 (50 nM; sc-61177, Santa Cruz Biotechnology) at a concentration of 50 nM; the rest of the transfection process was the same as described above.

### RNA and protein preparation

RNA and protein were extracted from cells at the same time using a Nucleospin® RNA/Protein Kit (cat# 740933, Macherey–Nagel GmbH & Co., Neumann-Neander-Str., Dueren, Germany) following the manufacturer’s instructions. The RNA concentration was measured using a NanoDropOne spectrophotometer (Thermo Fisher Scientific), and RT–PCR was performed with 2–5 μg of RNA. The protein concentration was measured using a BCA assay, and western blotting was performed with 20–50 µg of protein. Nphp 4 forward primer: 5′- CCTGCAGCGCGTGGATGT-3′, reverse primer: 5′- CTGGGGGTGGGAGGTGAAAG-3′; Nphp5 forward primer: 5′-TGGGAAGCTCAGAGTTAAAGAAAGT-3′, reverse primer: 5′- TGTGTAAGCTGGGAAATTGTAGACC-3′; Nphp6 forward primer: 5′-GCTCAGTGCTCTTCAGATGGATTC-3′, reverse primer: 5′-GTGTACTGCCGGATGAGGGACTT-3′; Nphp9 forward primer: 5′- TGCATCCGGGCCCTACTCAA-3′, reverse primer: 5′-GGTGGCCCTGCTCCCTGTG-3′.

### Western blotting

SDS–PAGE was performed by loading extracted protein samples on an 8–12% gel with a protein marker (PM2610, SMOBiO, Hsinchu City, Taiwan, R.O.C.). The separated proteins on the gel were electrotransferred to a PVDF membrane (AE-6667-P, Atto, Taito-ku, Tokyo, Japan) at 350 mA for 70 min. The membranes were blocked with 5% skim milk (232100, BD Difco, USA) in PBST and incubated with primary antibodies in 1% skim milk overnight at 4 °C. The next day, the membranes were incubated with HRP-conjugated secondary antibodies (ADI-SAB-300-J, goat anti-rabbit IgG (HRP conjugate) and ADI-SAB-100-J, goat anti-mouse IgG F (ab’)2 (HRP conjugate); Enzo Life Sciences, Farmingdale, NY, USA) in 2% skim milk for more than an hour at RT, and chemiluminescent signals were then detected using EzWestLumi plus reagent (2332637, Atto), a LAS3000 imager (Fujifilm) and an Amersham™ ImageQuant™ 800 biomolecular imager. The antibodies used to identify proteins during western blotting are described in Supplementary Table 1.

### Immunocytochemistry

Cells prepared on cover glasses were fixed with methanol at −20 °C for 10 min and blocked for 10 min-1 h with 1–5% permeabilization solution with BSA powder and 0.2% Triton X-100. Then, primary antibodies were added to the permeabilization solution and incubated overnight at 4 °C. Secondary antibodies and DAPI were added a day after incubation with primary antibodies. Finally, it was fixed to the slide glasses with the fluorescence mounting medium (S3023, Dako, Santa Clara, CA, United States). After a day at room temperature or 4 °C, images were acquired with a confocal fluorescence microscope (LSM 700, Zeiss, Carl-Zeiss-Strasse, Oberkochen, Germany). The antibodies used for ICC are described in Supplementary Table 2. For fluorescence intensity analysis, normalization was conducted by measuring the intensity of 3 to 5 signal-free spots in an image using the Zen program. Then, after the signal intensities were measured, the normalized value was determined by subtracting the signal-free fluorescence intensity value from the measured fluorescence intensity value.

### Mouse information and renal sampling

Three types of mouse lines were used in this study. The floxed TAZ (TAZ-floxed) mice were obtained from Catholic University and have been previously described^19,20^. The KSP-cre mice were also obtained from Catholic University and were generated from B6.Cg-Tg(Cdh16-cre)91Igr/J (Stock No: 012237) mice purchased from The Jackson Laboratory. HoxB7-cre mice were obtained from Chungnam National University. The genotyping primers are listed in Supplementary Table 3.

Mice (3, 8, 21, and 36 weeks old) were anesthetized with ether, and the body weights were measured. The kidneys were extracted and weighed and were then fixed with 10% neutral buffered formalin (FR2013-100-00, Biosesang). Subsequently, we sliced the kidneys into longitudinal sections to produce paraffin sections. H&E staining was performed at the Department of Laboratory Animal Resources, Yonsei University.

### Immunohistochemistry and Immunofluorescence

Paraffin-embedded slides of mouse kidney tissue were deparaffinized at 60 °C for 30 min and rehydrated with Histo-Clear II (HS-202, National Diagnostics, Atlanta, Georgia, USA) and 70%-100% ethanol. Then, the slides were placed into Borg Decloaker, RTU (BD1000G1, Biocare Medical, Pacheco, CA, USA), and antigen retrieval was performed in a TintoRetriever pressure cooker (BSB 7008, Bio SB, Santa Barbara, CA, USA), and blocked for 1 h with a blocking solution diluted with normal horse serum from a VECTASTAIN® Elite® ABC HRP Kit (PK-6200, Vector Labs, Burlingame, CA, USA). Primary antibodies were added to the blocking solution and incubated overnight at 4 °C. Secondary antibodies and DAPI were subsequently added and incubated for 2–3 h after several washing steps. Finally, the cover glasses were mounted to slides using fluorescence mounting medium (S3023, Dako). The antibodies used for immunofluorescence assays are described in Supplementary Table 2.

## Results

### Observation of cyst and cilia phenotypes in TAZ knockout mice

**Fig. 1:**
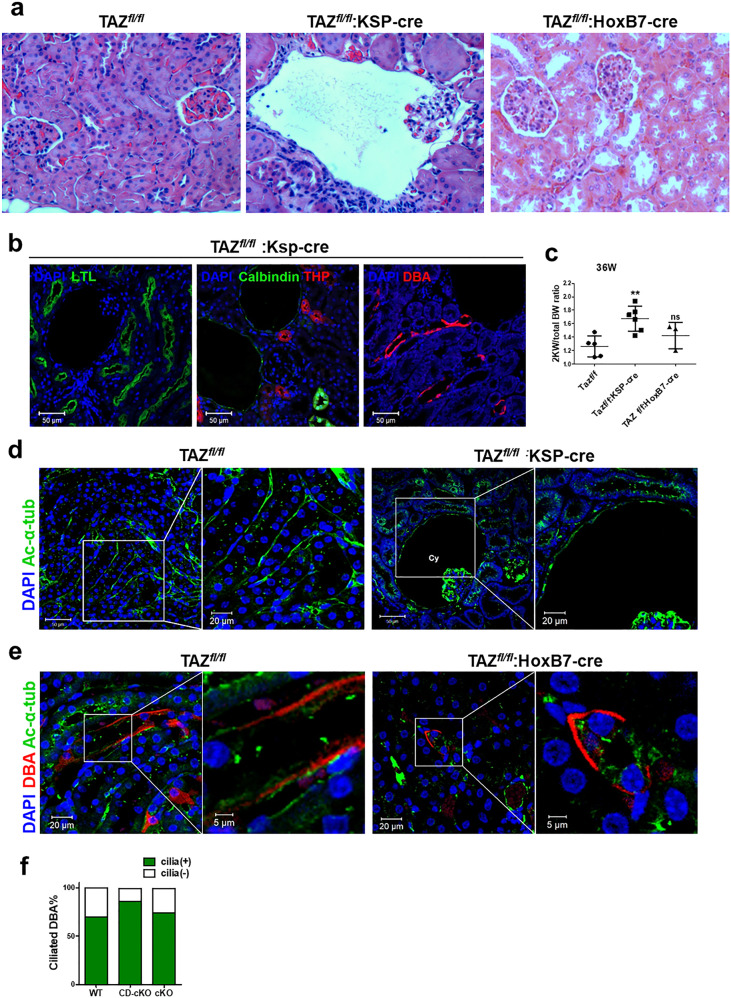
Cyst formation and cilia reduction in Taz-floxed:KSP-cre and Taz-floxed:HoxB7-cre mice. **a** Sections of renal tissues harvested from Taz-floxed, Taz-floxed:KSP-cre and Taz-floxed:HoxB7-cre mice at 36 weeks and stained using H&E. 400× magnification. **b** Fluorescence staining of tubule markers. LTL, proximal tubule; Calbindin, distal convoluted tubule; THP, ascending loop of Henle; DBA, collecting duct. 200× magnification. Images were acquired with a confocal microscope (Zeiss). **c** The ratio of the weight of the two kidneys to the total body weight (2KW/TBW) in WT, TAZ cKO, and TAZ CD-cKO mice at 36 weeks. **d** Staining of primary cilia labeled with acetylated α-tubulin. There were no cilia in cystic tubule cells. ‘Cy’ means kidney cyst. **e** Fluorescence staining with DBA and acetylated-α-tubulin in renal tissue sections from WT and TAZ CD-cKO mice; images were acquired with a confocal microscope (Zeiss). **f** Graph quantifying cilia-positive DBA labeling in WT, TAZ CD-cKO, and TAZ cKO mouse renal tissues. DBA signals in which at least one cilium was observed were classified as cilia-positive DBA signals, DBA signals without cilia were classified as cilia-negative DBA signals; and ratio is shown. Mean ± SD; **P < 0.01; n.s., not significant.

A previous study reported the occurrence of glomerular cysts in TAZ knockout mice^15^. Here, however, we used two different Cre mouse models with cre-specific TAZ knockout, namely, renal epithelial-specific (KSP)-cre mice and collecting duct-specific (HoxB7)-cre mice, to determine the novel role of TAZ in regulating renal primary cilia. As previously reported, renal tissue sections from TAZ^fl/fl^:KSP-cre (TAZ cKO) mice at 36 weeks of age showed mild cyst formation (Fig. 1a). Although not large, many cysts were found to originate in glomeruli. As the cysts in TAZ^fl/fl^:KSP-cre mice were observed at 36 weeks of age, TAZ^fl/fl^:HoxB7-cre (TAZ CD-cKO) mice were also investigated at the same stage, i.e., 36 weeks of age. Notably, no visible cysts were found in the renal tissue sections of the TAZ^fl/fl^:HoxB7-cre mice at 36 weeks (Fig. 1a). To determine whether cysts formed only in glomeruli, we performed immunofluorescence staining for various tubule markers (Fig. 1b). We found that other tubule markers (calbindin: distal tubule, Tamm-Horsfall glycoprotein (THP): ascending loop of Henle, *Dolichos biflorus* agglutinin (DBA): collecting tubule, *Lotus tetragonolobus* lectin LTL: proximal tubule) were not consistent with the occurrence of cysts. Thus, the renal cysts formation in TAZ cKO mice suggests that TAZ deficiency results in glomerular cysts only in the late developmental stage. We also calculated the ratio of the weight of the two kidneys to the total body weight (2 KW/TBW) as an indicator of cyst formation (Fig. 1c). The average 2KW/TBW ratio in the early stage was similar between TAZ^fl/fl^ (wild-type, WT) and TAZ^fl/fl^:KSP-cre (TAZ cKO) mice (Supplementary Fig. 1a), but the ratio in TAZ cKO mice was higher in the 36-week-old late stage than in the early stage, indicating that cyst formation was delayed. In addition, the average 2KW/TBW ratio in CD-cKO TAZ mice was not significantly different from that in WT mice. It is known that cre is not expressed in the renal glomerular epithelium in KSP-cre mice; however, we observed glomerular cysts, and we thus assessed cre recombinase expression in glomeruli^21^ (Supplementary Fig. 1b). Cre recombinase expression was not observed in wild-type mice but was confirmed in the proximal tubule, distal tubule, and collecting duct of TAZ cKO mice. Although cre was not expressed in glomerular podocytes, weak cre expression was confirmed in Bowman’s capsule cells surrounding the glomerulus (Supplementary Fig. 1c). We observed dilation of Bowman’s capsule in TAZ cKO mice with glomerular cysts of, as observed in a previous study^15^. Therefore, KSP-cre is predicted to induce cystic dilation of Bowman’s capsule epithelial cells surrounding the glomerulus.

As cystic kidneys are commonly associated with primary ciliary defects, we also checked for primary cilia in the cyst-lining epithelial cells of the renal tubules of TAZ cKO mice by immunofluorescence staining. We found that primary cilia were present in the epithelial tubule cells in wild-type mice but not on the surface of the cyst-lining cells in TAZ cKO mice (Fig. 1d). There was also no ciliary defect in the collecting duct cells in TAZ CD-cKO mice (Fig. 1e, f). In summary, although TAZ deficiency did not play a significant role in the collecting duct, it certainly played a role in the whole kidney and could be considered particularly important in cilia formation in renal glomeruli.

### Differential localization and expression of TAZ in each renal tubule

**Fig. 2:**
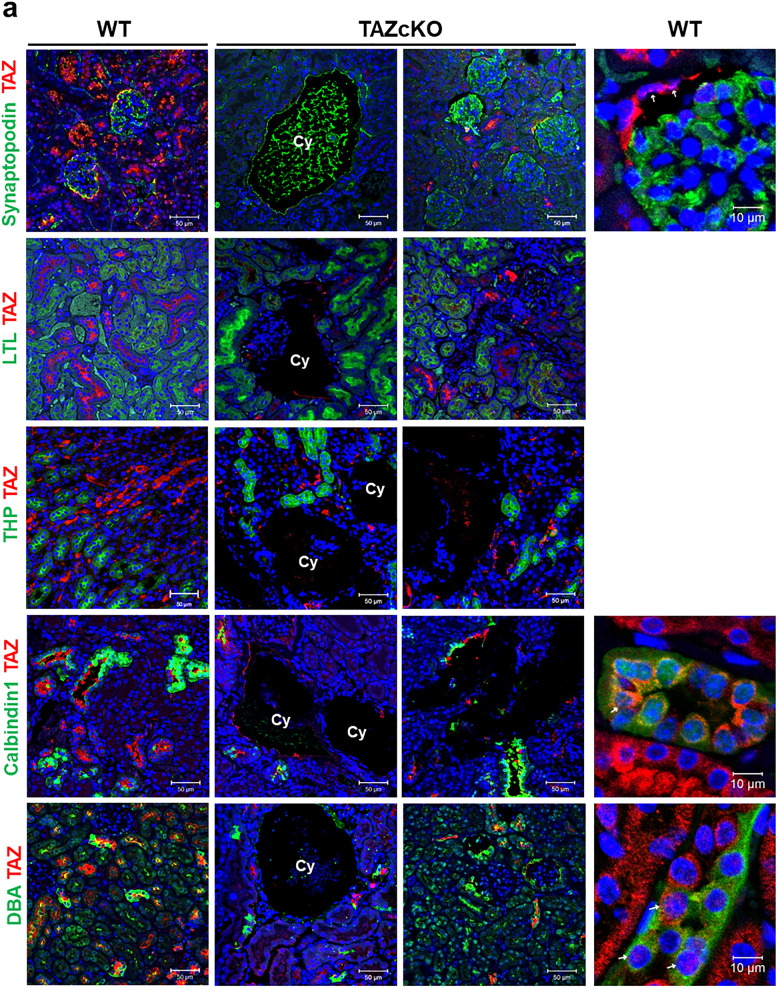
Different localization of TAZ in specific renal tubules. **a** Fluorescence staining of TAZ with tubule-specific markers in renal tissues from 36-week-old WT and Taz cKO mice. Synaptopodin, renal podocytes; LTL, proximal tubule; THP, ascending loop of Henle; Calbindin, distal convoluted tubule; DBA, collecting duct. ‘Cy’ means kidney cyst. 200× magnification and 0.5× reverse zoom (right). The magnified images show the cytosolic/nuclear localization of TAZ in each renal tubule. The cells indicated by the white arrows were observed to have nuclear TAZ expression.

As a ciliopathy phenotype was observed in whole kidney-specific TAZ-deficient (TAZ cKO) mice but collecting duct-specific TAZ deficiency (i.e., Taz CD-cKO) showed little effect, we hypothesized that the role of TAZ in each renal tubule cell type is different. First, the localization of TAZ expression was confirmed in each renal tubule in wild-type (Taz^fl/fl^) mice (Fig. 2a). There was no colocalization of TAZ with markers of the proximal tubule (LTL) and the ascending loop of Henle (THP), whereas partial TAZ expression was found in the collecting duct (DBA), distal tubule (Calbindin 1), and the surrounding glomerular podocytes (synaptopodin). The differential expression levels of TAZ in each renal tubule did not provide insights into its role but suggested that it may be less important in tubules where its expression is low expression. We then evaluated TAZ expression in TAZ cKO mouse renal tissues. Although the cre recombinase system was used, some TAZ expression remained, but no TAZ expression was observed at the sites of cyst development. The cytosolic/nuclear localization of TAZ was evaluated through enlarged images of the renal tubules expressing TAZ, i.e., glomeruli, distal tubules, and collecting ducts (Fig. 2a, right). In podocytes labeled with synaptopodin, almost no TAZ expression was observed, but expression of both cytosolic and nuclear TAZ were observed in Bowman’s capsule epithelial cells surrounding the glomerulus, and cytosolic TAZ showed a relatively stronger signal. In the calbindin-labeled distal tubule, TAZ was mostly localized in the cytosol, and DBA-labeled collecting ducts also showed both cytosolic and nuclear TAZ, but not all collecting ducts showed TAZ expression.

Subsequently, we confirmed the level of TAZ expression under physiological conditions in each tubule through evaluation of cell lines, namely, TKPTS (proximal tubule), mIMCD (collecting duct), and SV40MES13 (glomeruli) (Supplementary Fig. 2a). The expression levels of YAP/TAZ differed in each cell type. The expression level of YAP was higher than that of TAZ in TKPTS and mIMCD cells. In particular, YAP expression in SV40MES13 cells was very low compared to that in the other two cell lines, and TAZ expression was very high. In addition, we verified phosphorylation of LATS and TAZ in each renal cell type under physiological conditions (Supplementary Fig. 2b). LATS expression was almost absent in mIMCD cells, and the presence of dephosphorylated LATS was confirmed in TKPTS cells. However, the levels of phosphorylated LATS and phosphorylated TAZ were high in SV40MES13 cells, suggesting that TAZ has a cytosolic function mediated by phosphorylated LATS in SV40MES13 cells.

Therefore, the appearance of glomerular cysts in the cKO mouse model and the almost negligible presence in other tubules may occur because TAZ expression differs by renal tubule type.

### TAZ-specific control of ciliogenesis

**Fig. 3:**
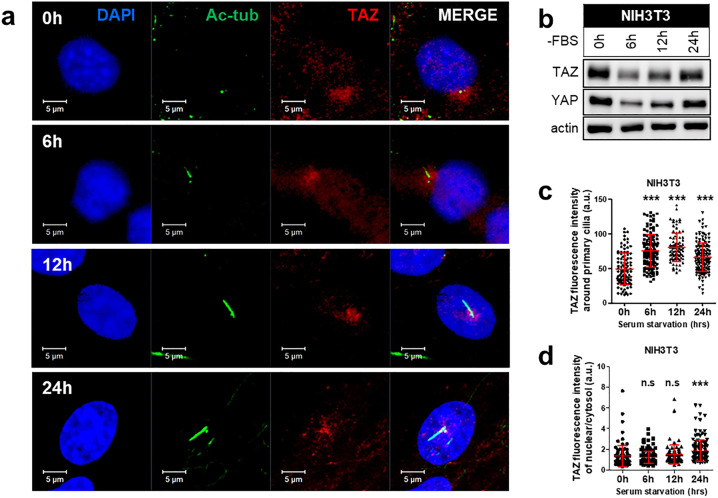
Changes in TAZ localization during primary cilia formation. **a**–**d** Observations of TAZ localization in NIH3T3 cells upon serum starvation. Serum was removed 2 d after seeding, and cells were finally harvested at each indicated time point (0, 6, 12, and 24 h) after FBS withdrawal. **a** Acetylated α-tubulin-labeled primary ciliary axoneme. Images were acquired at 400× magnification. **b** Comparison of YAP/TAZ expression under serum starvation conditions by immunoblot analysis. **c** The graph shows the fluorescence intensity of TAZ around primary cilia at 0, 6, 12, and 24 h after serum withdrawal. **d** The ratio of the nuclear TAZ fluorescence intensity to the cytosolic TAZ fluorescence intensity over a time-course after serum starvation. The mean fluorescence intensity value was measured with the ZEN blue edition image processing program. Mean ± SD; ***P < 0.001; n.s., not significant.

Based on previous observations, we hypothesized that TAZ might affect ciliogenesis in renal cells. We investigated TAZ alterations during cilia formation and identified changes in TAZ colocalization with cilia under serum starvation conditions in vitro. The increase in TAZ expression with increased time under serum starvation conditions may indicate that it affects cilia formation^22^. The locations of primary cilia and TAZ were determined by immunofluorescence staining using NIH3T3 cells because they do not differentiate into any cell type and are commonly used in ciliary studies (Fig. 3a). The conditions for ciliogenesis were maintained for 24 h under serum starvation and were observed at serial time points. At the early time point of serum starvation (6 h), we found that TAZ was largely distributed in the cytoplasm. As the serum starvation time increased, TAZ gradually accumulated in the nucleus, and its expression was concentrated at the ciliary base. Total YAP/TAZ expression decreased during serum starvation at 6 h and 12 h and was restored at 24 h, when primary cilia were fully formed (Fig. 3b). We digitized images to determine the TAZ fluorescence intensity around the primary cilia, nucleus, and cytosol using the blue edition of Zeiss ZEN software and normalized it to the background intensity of TAZ. The TAZ intensity around primary cilia increased with time (Fig. 3c), and the ratio of nuclear TAZ to cytosolic TAZ increased at 24 h (Fig. 3d). Thus, these data imply that TAZ may play a role in the formation of primary cilia.

Next, we investigated whether TAZ has different effects on each renal tubule cell type, as it showed different phenotypes in wild-type kidney tissues. We induced primary ciliogenesis in the mIMCD, SV40MES13, and TKPTS cell lines, and 24 h after the removal of fetal bovine serum (FBS), we determined that primary cilia were almost fully assembled in the last 24 h of serum starvation. We identified differences in TAZ expression during ciliogenesis between cell types (Supplementary Fig. 2c). After 24 h of serum starvation, the expression of YAP/TAZ decreased in mIMCD and SV40MES13 cells, similar to the observations in NIH3T3 cells. However, TKPTS showed increased protein expression after 6 h of serum starvation, followed by decreased expression. In mIMCD, SV40MES13, and NIH3T3 cells, TAZ expression decreased initially at 6 h of serum starvation and then recovered at 24 h, when cilia were fully assembled, but its expression trend was the opposite in TKPTS cells. These results suggest that TAZ localized near primary cilia participates in cilia formation and that this process is dependent on the type of renal tubule cell.

### Opposite roles of YAP and TAZ in ciliogenesis depending on the renal tubular cell type

To investigate the role of TAZ in cilia formation in each renal tubule, we performed loss-of-function experiments using siRNAs in vitro. Previous studies on YAP/TAZ and ciliogenesis suggested that these two proteins showed opposite effects: YAP mainly inhibited cilia formation^16,23^, and only TAZ played a role in ciliogenesis^15,18^. Thus, we knocked down TAZ and YAP independently in our experiment to study their roles in cilia formation.

**Fig. 4:**
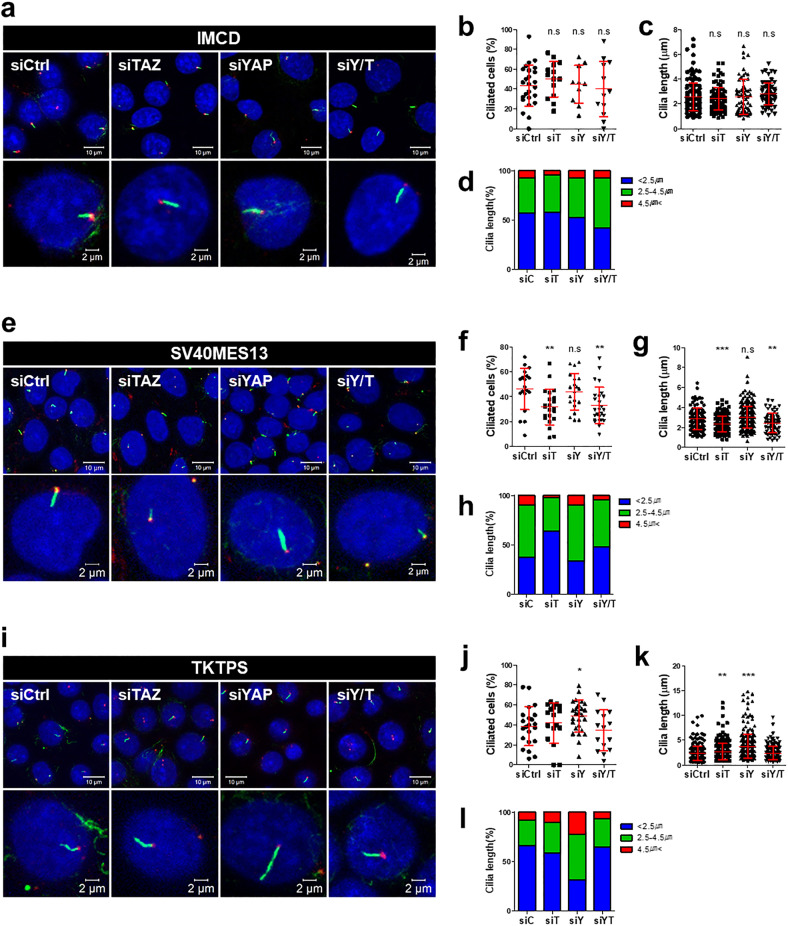
Different roles of YAP and TAZ in cilia formation in multiple types of renal tubular cells. **a**–**l** Observations of fluorescently labeled primary cilia (antibodies targeting Ac-α-tubulin and pericentrin were used for the axoneme and basal body, respectively) after silencing of either YAP or TAZ in three different types of cells—mIMCD, SV40MES13, and TKPTS. Here, siRNA was used at 20 nM for single transfection and 10 nM each for double transfection; cells were then serum starved for at least 24 h. **a**, **e**, **i** Each cell at 400× magnification and 1.5× magnification. SV40MES13 and TKPTS cells at 630× magnification with an oil objective. The images in each lower panel show a higher magnification cell image with a representative cilia length. **b**, **f**, **j** The graphs show the ratio of the number of ciliated cells to the number of DAPI-stained nuclei per image. **c**, **g**, **k** Cilia lengths were measured from the basal body to the ciliary tip. **d**, **h**, **l** The graphs show the proportions of cilia lengths in three ranges: <2.5 µm, >2.5 μm and <5 μm, and >5 μm. Mean± SD, *P < 0.05, **P < 0.01, ***P < 0.001.

We generated three different types of renal tubular cells and conducted experiments under the same conditions. Cells were transfected with siRNAs targeting either *Yap* or *Taz*, and the medium was replaced with FBS-free medium for at least 24 h. The levels of YAP and TAZ knockdown using siRNA were confirmed by immunoblotting (Supplementary Fig. 3a–c). To compare cilia formation under each condition, we determined the number of ciliated cells and measured the length of cilia. Cilia were well assembled in mIMCD cells after serum starvation; however, TAZ knockdown cells showed no significant difference in ciliary assembly relative to control cells (Fig. 4a). Regardless of functional loss of only YAP or YAP/TAZ simultaneously, the average ciliated cell rate and cilia length were similar to those in control cells (Fig. 4b, c). In addition, when the length of cilia was categorized as <2.5 µm, 2.5–4.5 µm, or >4.5 µm and then quantified, there were some differences; the proportion cilia with a length of <2.5 µm was reduced and the proportion of cilia with a length of 2.5–4.5 µm was increased in YAP/TAZ-silenced cells, but there were no major changes (Fig. 4d). In summary, consistent with the previous in vivo results in Taz CD-cKO mice, TAZ deficiency in the collecting duct did not affect cilia formation.

The findings of the following experiments conducted in SV40MES13 cells were different from those conducted in mIMCD cells. Interesting results were expected from this experiment because of the glomerular cysts and the reduction in cilia on cyst-lining cells in 36-week-old Taz cKO mice. We found that both YAP and TAZ were successfully targeted and that serum starvation induced cilia formation (Supplementary Fig. 3b). Notably, TAZ knockdown in SV40MES13 cells decreased the ciliated cell rate and cilia length (Fig. 4e–g). A considerable reduction in cilia was also observed when YAP and TAZ were simultaneously knocked down. In contrast, when only YAP was knocked down, the ciliated cell rate and cilia length were not affected (Fig. 4e). The proportion of cilia shorter than 2.5 µm increased when TAZ expression decreased (Fig. 4h). These results demonstrated that TAZ positively regulates cilia formation in glomeruli, independent of YAP.

Finally, the same experiment was conducted in TKPTS cells. Remarkably, the decrease in YAP expression caused an increase in the proportion of ciliated cells and the cilia length (Fig. 4i–k). In particular, the proportion of cilia with a length of more than 4.5 µm was increased significantly in TKPTS cells compared with other tubule cell types, indicating that YAP negatively regulates cilia formation in TKPTS cells (Fig. 4l). However, TAZ knockdown did not affect ciliogenesis in TKPTS cells, and even when YAP and TAZ were knocked down simultaneously, the ciliary phenotype was the same as that in control cells. In conclusion, the functions of TAZ not only differ in various renal tubule cells but also distinctively regulate ciliogenesis. It is also conceivable that YAP and TAZ play opposite roles in ciliary regulation in each renal tubule cell.

### Validation of ciliary genes that change with decreased TAZ expression

We investigated ciliary proteins under conditions equivalent to those used for investigating ciliary phenotypes in glomerular cells to determine whether primary cilium formation is altered by TAZ. We first observed IFT protein transport on the primary cilium axoneme. IFT proteins migrate through the primary cilium axoneme to regulate the abilities of primary cilia and play a role in controlling cilia assembly by riding on cilia. Deficiencies in IFT protein expression lead to defects in cilia assembly^2^. To investigate ciliary defects, we investigated the changes in IFT protein expression induced by YAP/TAZ silencing.

**Fig. 5:**
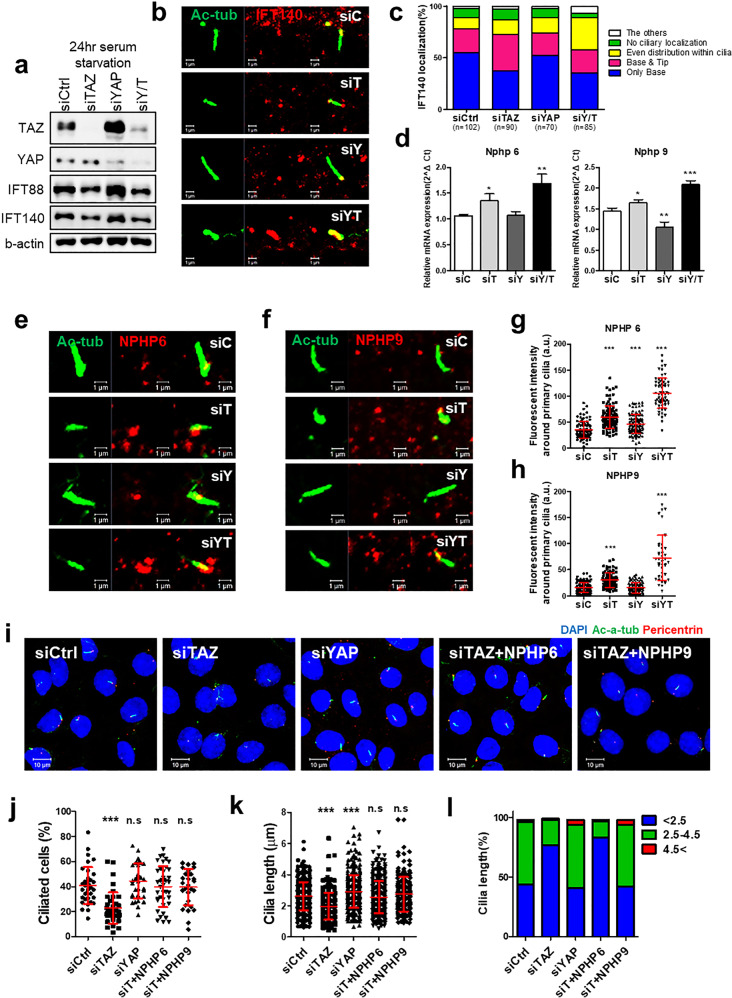
IFT140, NPHP6, and NPHP9 changed with decreased TAZ expression. **a** Western blots showing IFT88 and IFT140 expression in cells with YAP or TAZ silencing and subjected to 24 h of serum starvation. **b** Immunocytochemical analysis of IFT140 (red) and the ciliary basal body (green) in SV40MES13 cells with YAP or TAZ knockdown and subjected to 24 h of serum starvation. **c** The localization of IFT140 in primary cilia was classified into 4 categories: localized only at the ciliary base, localized at both the base and tip, evenly distributed throughout cilia, and not localized in cilia. **d** *NPHP6* and *NPHP9* mRNA expression changed in SV40MES13 cells with YAP/TAZ knockdown and subjected to 24 h of serum starvation. **e**, **f** Accumulation of NPHP6 and NPHP9 (red) in primary cilia (green) under identical conditions in SV40MES13 cells. **g**, **h** The graph shows the fluorescence intensities of NPHP 6 and 9 localized around primary cilia; fluorescence intensities were measured by ZEN blue edition imaging software (Zeiss). **i**–**l** Primary cilia recovery in cells with TAZ silencing and knockdown of either NPHP 6 or 9 after 24 h of serum starvation. **i** Fluorescence images showing primary cilia in cells with knockdown of TAZ or YAP or double knockdown of TAZ and NPHP 6 or 9 after 24 h of serum starvation. **j** The graphs show the ratio of the number of ciliated cells to the number of DAPI-stained nuclei per image. **k** Primary cilia lengths were measured by the ZEN black edition program. **l** The graphs show the proportions of cilia lengths in three ranges: <2.5 μm, 2.5 μm to 5 μm, and >5 μm. a.u. means arbitrary unit. Mean ± SD, *P < 0.05, **P < 0.01, ***P < 0.001.

Western blot analysis confirmed that the expression of IFT88 and IFT140 decreased with TAZ or YAP/TAZ knockdown, and we subsequently observed their ciliary localization (Fig. 5a). IFT140 in the IFT-A complex was retrogradely transported from the ciliary tip to the ciliary base and showed a change in ciliary localization. IFT140 localization in cilia was classified into the following four categories: localized only at the ciliary base, localized at both the base and tip, evenly distributed throughout primary cilia, and not localized in cilia (Fig. 5b, c)^24,25^. Over half of the expressed IFT140 was localized only in the ciliary base. This pattern was similar to that seen in control cells when YAP expression was knocked down. In the case of decreased TAZ expression, the proportion of IFT140 localized only in the base decreased, but the proportion of IFT140 localized in the tip increased further. YAP/TAZ silencing led to an increase in the proportion of IFT140 localized throughout cilia. Thus, based on the level of TAZ expression in addition to YAZ expression, IFT140 was distributed throughout cilia or at the ciliary tip.

Conversely, IFT88 in the IFT-B complex was anterogradely transported from the ciliary base to the tip and showed a slight change in intensity or localization due to a decrease in TAZ expression (Supplementary Fig. 4a, b). In the case of YAP/TAZ double knockdown, the fluorescence intensity of IFT88 was increased, and IFT88 localization on primary cilia showed an increased proportion of even distribution (Supplementary Fig. 4c). This even distribution maybe because of the shorter cilia length. In the case of decreased YAP expression, IFT88 did not accumulate on either side of the tip or base but was generally distributed evenly across cilia relative to its distribution in control cells. This corroborates the finding that a decrease in YAP expression in glomerular cells did not affect the reduction in cilia length.

Subsequently, we selected another ciliary gene related to TAZ, the *NPHP* gene, which showed significant changes during ciliogenesis under serum starvation in SV40MES13 cells with YAP/TAZ knockdown^26,27^. We selected *NPHP 4, 5, 6*, and *9*, which showed changes in mRNA expression only with reduced TAZ expression (Fig. 5d and Supplementary Fig. 5a), and assessed their localization and intensity near primary cilia via immunofluorescence staining. NPHP4 and NPHP5 showed an increased fluorescence intensity near primary cilia when both YAP and TAZ were knocked down, but there were no noticeable differences compared to control cells when only TAZ was knocked down (Supplementary Fig. 5b, c). Therefore, NPHP4 and NPHP5 were not selected because they are less relevant for TAZ-mediated regulation of cilia. However, the fluorescence intensity throughout the cell, particularly near the shortened primary cilia in NPHP6 and NPHP9 cells, increased in the same pattern, with increased mRNA expression dependent on the decrease in TAZ expression (Fig. 5e–h).

Next, we observed changes in primary cilia when NPHP6 and NPHP9 were knocked down in cells with TAZ deficiency (Fig. 5i–l). The experimental protocol was the same as that described before, and only *Nphp6* and *Nphp9* knockdown cells were included. The knockdown efficiency of the NPHP6 and NPHP9 siRNA constructs was confirmed via mRNA expression analysis (Supplementary Fig. 5d). Surprisingly, in cells with double knockdown of TAZ and either NPHP6 or NPHP9, the ciliated cell rate and cilia length were significantly restored (Fig. 5j, k). The cilia length distribution did not change significantly in the case of double knockdown of TAZ and NPHP6, even resembling that in cells with TAZ knockdown, but showed values similar to those in control cells when both TAZ and Nphp9 were knocked down (Fig. 5l). In TAZ/NPHP9 double knockdown cells, the percentage of cilia with a length of <2.5 µm was <50%, and the percentage of cilia with a length of >4.5 µm was greater than 50%, similar to the percentages in control cells.

*NPHP9* mRNA expression analysis confirmed that the increase due to TAZ knockdown was also balanced to a certain level via double knockdown of TAZ and NPHP9 and triple knockdown of YAP/TAZ and NPHP9 (Supplementary Fig. 5d). Primary ciliary recovery was also verified by triple knockdown of YAP/TAZ and either NPHP6 or NPHP9 (Supplementary Fig. 5e–h). In summary, the absence of TAZ caused ciliary defects in SV40MES13 cells but reconfirmed the anomalous distribution of IFT140 on cilia. In addition, TAZ deficiency increased NPHP6 and NPHP9 localization around primary cilia, suggesting that reduced expression of NPHP6 and NPHP9 results in a ciliary recovery effect.

Therefore, we established that cilia are shortened or decreased in number by TAZ in SV40MES13 glomerular cells and confirmed the possibility of restoring the cilia length. The detailed mechanism by which NPHP regulates ciliogenesis (Fig. 5) and whether NPHP is regulated directly by TAZ requires further research. However, by confirming that ciliary genes are coregulated by changes in TAZ expression, our findings suggest that TAZ and its related proteins may regulate cilia formation. We also analyzed how TAZ regulates cilia formation in renal tubules and the relationship between YAP and TAZ in ciliary assembly. To the best of our knowledge, this study is the first to suggest the differential roles of TAZ in cilia formation depending on the type of renal tubule. In addition, our results suggest the independent and contradictory roles of YAP and TAZ; however, this finding needs to be validated through detailed analysis of the mechanisms and application of disease models.

## Discussion

Primary cilia are organelles that deliver external signals to cells and are important because they are involved in various signaling mechanisms. Renal primary ciliary defects are caused by genetic mutations and are associated with renal tubular cyst formation. Recent studies have reported that Hippo signaling regulates cilia, and some other studies have suggested that YAP/TAZ regulate ciliogenesis. However, no study has specifically described the role of TAZ in cilia formation independent of YAP^16,23^. In this study, we elucidated the role of TAZ in ciliogenesis in different types of renal tubule cells and its primary regulatory role in glomeruli.

Cilia with a mild cystic phenotype and cyst-lining cells were reduced in -36-week-old (late-stage) mice with whole kidney-specific TAZ deficiency. However, no cystic phenotype was observed in mice with collecting duct-specific TAZ deficiency, and the expression of TAZ was different in each renal tubule cell line. The differential expression of TAZ in different types of renal tubules was responsible for the different cystic phenotypes in the mouse models. Evidently, protein expression is not an indicator of protein function, but it could be expected that in the tubules without TAZ expression, the function of TAZ was negligible. In addition, we considered not only the expression pattern but also the localization of TAZ. TAZ colocalized in the distal tubule was mostly cytosolic, and both nuclear and cytoplasmic TAZ was observed in Bowman’s capsule and the collecting duct. Different renal tubule cell types showed different expression levels of the YAP and TAZ proteins in vivo and in vitro, and the patterns of phosphorylated LATS and TAZ were also different. Therefore, we speculate that cytosolic TAZ induces TAZ-specific ciliary regulation in SV40MES13 cells.

We also found different roles of YAP/TAZ in primary cilia formation in each renal cell type. YAP and TAZ did not regulate ciliogenesis in collecting duct cells or IMCD cells. In contrast, cilia formation was regulated by TAZ independent of YAP in SV40MES13 glomerular cells, as confirmed in TAZ cKO mice, and TAZ deficiency reduced cilia formation. In TKPTS proximal tubule cells, unlike the other cell lines, both the length and number of primary cilia increased under YAP deficiency. Because of the low TAZ level in normal renal proximal tubule tissue, it was hypothesized that the role of TAZ in ciliary regulation in the proximal tubule is minimal. According to published reports, a decrease in TAZ expression tends to decrease the number of cilia, and a decrease in YAP expression tends to increase the number of cilia^15,16,18,23^. Thus, we identified the potential roles of YAP and TAZ in ciliogenesis.

Cilia were shortened or decreased in number by Taz in the glomerulus, and thus, we also assessed other ciliary genes. IFT140 regulates ciliary entry while acting as a gatekeeper in the transition zone^28,29^. It was reconfirmed that ciliary defects occur due to a decrease in TAZ expression and that IFT140 transported through cilia does not function at the base but accumulates at the tip or axoneme. IFT88 is also a very important protein for generating primary cilia, and its expression was decreased with TAZ knockdown but showed no significant change in localization to the ciliary axoneme. However, notably, IFT88 was observed outside shortened cilia, as if it reproduced the shape of primary cilium (Supplementary Fig. 4a).

Furthermore, we also predicted that the NPHP protein, which causes nephronophthisis in the setting of mutation or Hippo signaling dysregulation, is associated with TAZ and cilia. We screened the expression of more than 20 *NPHP* genes at the mRNA level^30^, selected four genes that might be regulated by TAZ knockdown in SV40MES13 cells, and identified two significantly regulated *NPHP* genes through immunocytochemistry. In the context of reduced TAZ expression, *NPHP6* and *NPHP9* showed increased intensity around primary cilia and throughout cells. Increased NPHP6 or NPHP9 expression rarely decreased the cilia length, but many studies have suggested that cilia are elongated in cells with mutations in Nphp6 (Cep290) or Nphp9 (Nek8)^31–35^. In addition, silencing of TAZ and either NPHP6 or NPHP9 led to the recovery of cilia length to a certain degree. Confirming whether TAZ directly or indirectly regulates IFT and NPHP protein expression requires further investigation, but its relevance is clear, because NPHP9 is known to directly regulate the nuclear transport of TAZ^36^. It is possible that LATS and TAZ were phosphorylated in SV40MES13 cells and that ciliary recovery was achieved by reducing the elevated expression of NPHP9; therefore, the cytosolic function of TAZ is expected to play an important role in regulating ciliogenesis in glomeruli.

In summary, YAP and TAZ are paralogs, but YAP has more domains than TAZ. Many studies have hypothesized that YAP might be more important than TAZ. However, a recent study found that YAP and TAZ can play different roles in ciliogenesis and cyst formation, and our results suggested that TAZ may regulate primary cilia in cooperation with NPHP in the glomerulus. The mechanism by which TAZ regulates cilia formation independent of YAP needs to be further studied, and the possibility that TAZ deficiency-induced glomerular cysts may regulate cyst formation through ciliary recovery also needs to be discussed. A previous study found that YAP expression is known to increase with a decrease in NPHP9 expression, and the cyst formation phenotype caused by the decrease in NPHP9 was observed to improve with YAP inhibition^37^. Although the referenced result was generated through changes in YAP expression, there is a possibility that NPHP restores glomerular cyst formation in TAZ cKO mice, which could be investigated if experiments are performed to determine the relationship among TAZ, NPHP and cyst formation in the glomerulus.

## Supplementary information

Supplementary figures, tables
