# Supplementary material for: Reduced expression of TAZ inhibits primary cilium formation in renal glomeruli
Source: Exp Mol Med. 2022 Feb 17;54(2):169–79. doi: 10.1038/s12276-022-00730-2 (PMC8894487; doi:10.1038/s12276-022-00730-2)
Supplement: Supplementary file 1 — Supplementary figures, tables [file 12276_2022_730_MOESM1_ESM.pdf]

Supplementary Tables

Supplementary Table 1. The list of primary antibodies using western blotting

Protein	company	product	catalog
phos-YAP	cell signaling	Phospho-YAP (Ser127) (D9W2I) Rabbit mAb	13008s
phos-TAZ	cell signaling	Phospho-TAZ (Ser89) (E1X9C) Rabbit mAb	59971s
active YAP	abcam	Anti-active YAP1 antibody [EPR19812]	ab205270
YAP/TAZ	cell signaling	YAP/TAZ (D24E4) Rabbit mAb	8418s
Phospho-LATS1	cell signaling	Phospho-LATS1 (Thr1079) (D57D3) Rabbit mAb	8654
LATS1	cell signaling	LATS1 (C66B5) Rabbit mAb	3477
IFT88	proteintech	IFT88 Rabbit Polyclonal antibody	13967-1-AP
IFT140	proteintech	IFT140 Rabbit Polyclonal antibody	17460-1-AP
Primary cilia	sigma	Monoclonal Anti-Tubulin, Acetylated antibody produced in mouse	T6793-.2ML
	proteintech	ARL13B Antibody(Rabbit Polyclonal)	17711-1-AP
basal body	sigma	Monoclonal Anti-γ-Tubulin antibody produced in mouse	T6557
	abcam	Anti-Pericentrin antibody - Centrosome Marker	ab4448
β-actin	BETHYL	Rabbit anti-Cytoskeletal Actin Antibody	A300-491A

Supplementary Table 2. The list of antibodies using immunofluorescence staining

Protein	company	product	catalog
TAZ	abcam	Anti-TAZ antibody -rabbit	ab84927
YAP	abcam	Anti-YAP1 antibody [EP1674Y] -rabbit	ab52771
Cre	novus	Cre Antibody	NB100-56133
ascending loop	abcam	Anti-Claudin 1 antibody	ab15098
	santa cruz	THP (B-2)	sc-271022
glomerulus	progen	anti-Synaptopodin mouse monoclonal, G1D4, supernatant concentrate	65294
distal tubule	sigma	Monoclonal Anti-Calbindin-D-28K antibody produced in mouse	C9848 (clone CB-955)
basal body	abcam	Anti-Pericentrin antibody - Centrosome Marker	ab4448
	sigma aldrich	Monoclonal Anti-γ-Tubulin antibody produced in mouse	T6557
Primary cilia	sigma	Monoclonal Anti-Tubulin, Acetylated antibody produced in mouse	T6793-.2ML
	proteintech	ARL13B Antibody(Rabbit Polyclonal)	17711-1-AP
IFT88	proteintech	IFT88 Rabbit Polyclonal antibody	13967-1-AP
IFT140	proteintech	IFT140 Rabbit Polyclonal antibody	17460-1-AP
Nphp4	proteintech	NPHP4 Rabbit Polyclonal antibody	13812-1-AP
Nphp5	proteintech	NPHP5,IQCB1 Antibody	15747-1-AP
Nphp6	proteintech	CEP290 Rabbit Polyclonal antibody	22490-1-AP
Nphp9	mybiosource	Rabbit NEK8 Polyclonal Antibody	MBS126251
nucleus	sigma	DAPI for nucleic acid staining	D9542
Secondary fluorescent antibodies	invitrogen	Alexa Fluor® 488, IgG (H+L) Highly Cross-Adsorbed Goat anti-Mouse	A11029
	invitrogen	Alexa Fluor® 488, IgG (H+L) Highly Cross-Adsorbed Goat anti-Rabbit	A11034
	invitrogen	Alexa Fluor® 594, IgG (H+L) Highly Cross-Adsorbed Goat anti-Rabbit	A11037
	invitrogen	Alexa Fluor® 594, IgG (H+L) Highly Cross-Adsorbed Goat anti-Mouse	A32742

Supplementary Table 3. The primers of mouse genotyping

Gene	Primer	Sequence
TAZ floxed	TAZ forward	5'-GGCTTGTGACAAAGAACCTGGGGCTATCGTAG-3'
	TAZ reverse	5'-CCCACAGTTAAATGCTTCTCCCAAGACTGGG-3'
KSP-cre	KSP 9074	5'-AGGCAAATTTTGGTGTACGG-3'
	KSP 9808	5'-GCAGATCTGGCTCTCCAAAG-3'
	KSP 8744	5'-CAAATGTTGCTTGTCTGGTG-3'
	KSP 8745	5'-GTCAGTCGAGTGCACAGTTT-3'
HoxB7-cre	HoxB7 forward	5'-GCGGTCTGGCAGTAAAAACTATC-3'
	HoxB7 reverse	5'-GTGAAACAGCATTGCTGTCACTT-3'

Supplementary Figures

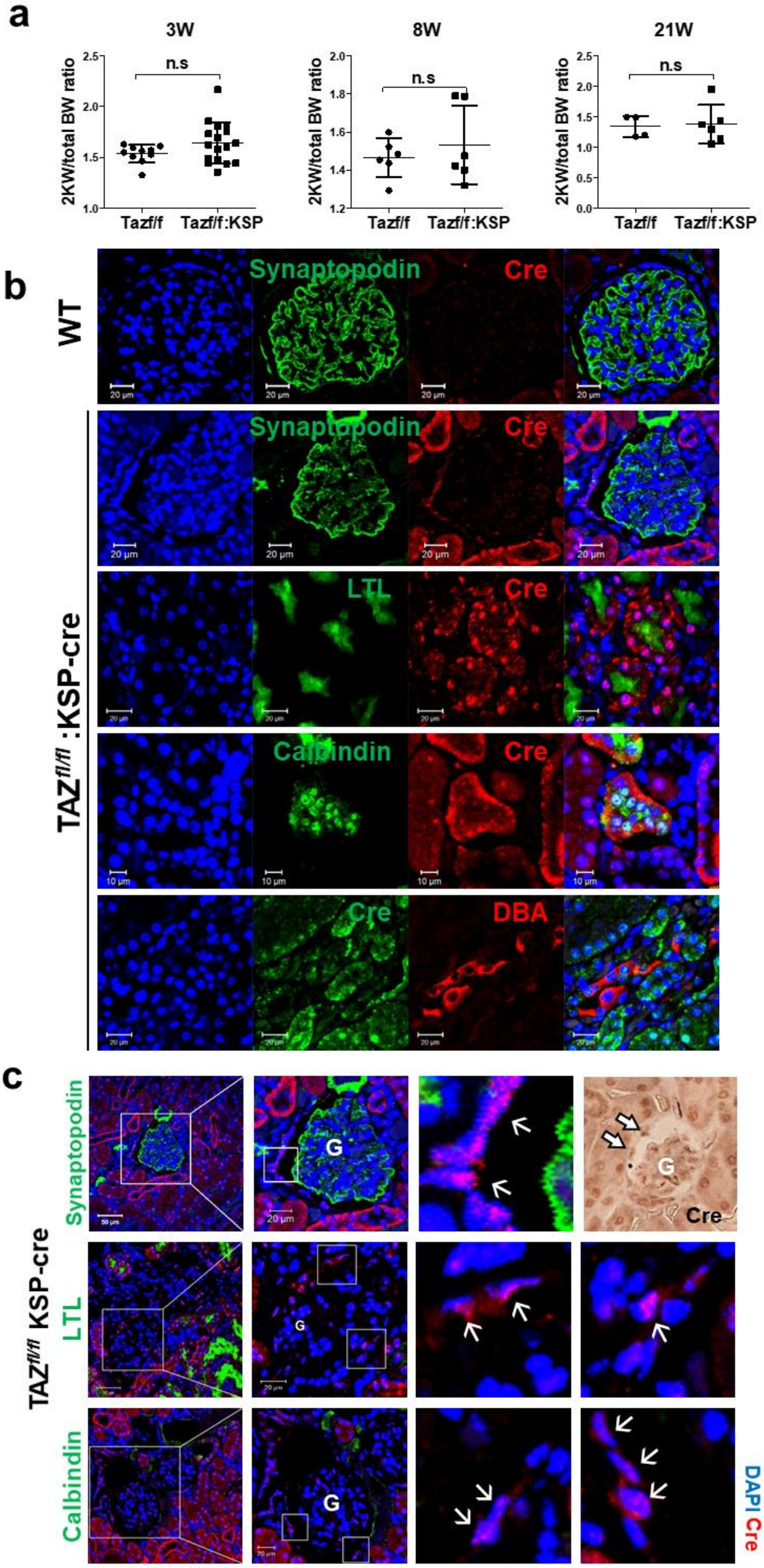

a
3W
8W
21W
n.s
2KW/total BW ratio
Tazf/f
Tazf/f:KSP
b
WT
TAZ fl/fl :KSP-cre
Synaptopodin
Cre
LTL
Calbindin
DBA
20 μm
10 μm
c
TAZ fl/fl KSP-cre
Synaptopodin
LTL
Calbindin
G
DAPI Cre

Supplementary Fig 1. Little differences in 2KW/TBW ratio between WT and TAZ-cKO at early stages and Cre-recombinase expression with KSP-cre mice.

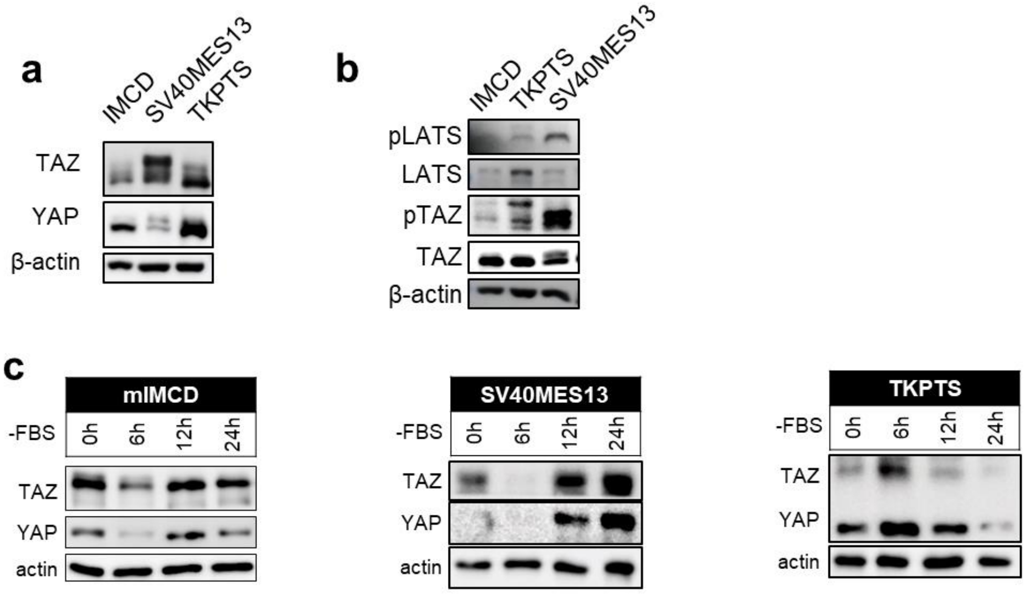

Supplementary Fig 2. YAP and TAZ expression on renal tubule cells under standard condition and during serum starvation.

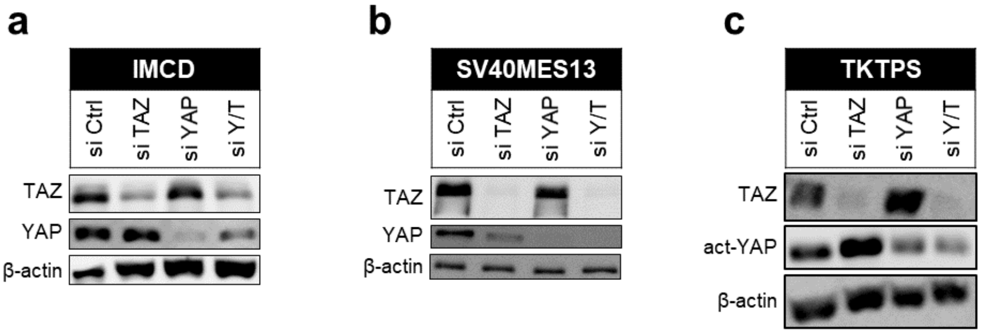

Supplementary Fig 3. Protein expressions under siRNA transfection.

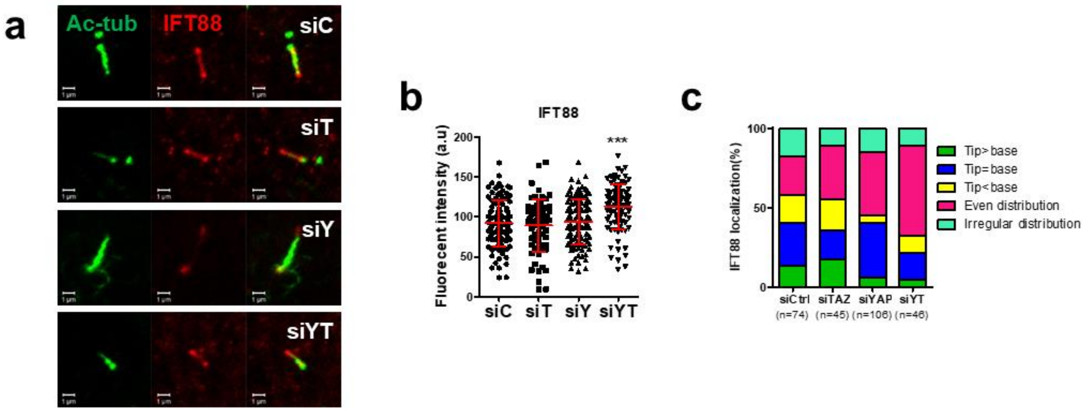

Supplementary Fig 4. No significant changes IFT88 expression under YAP and TAZ decrease in SV40MES13.

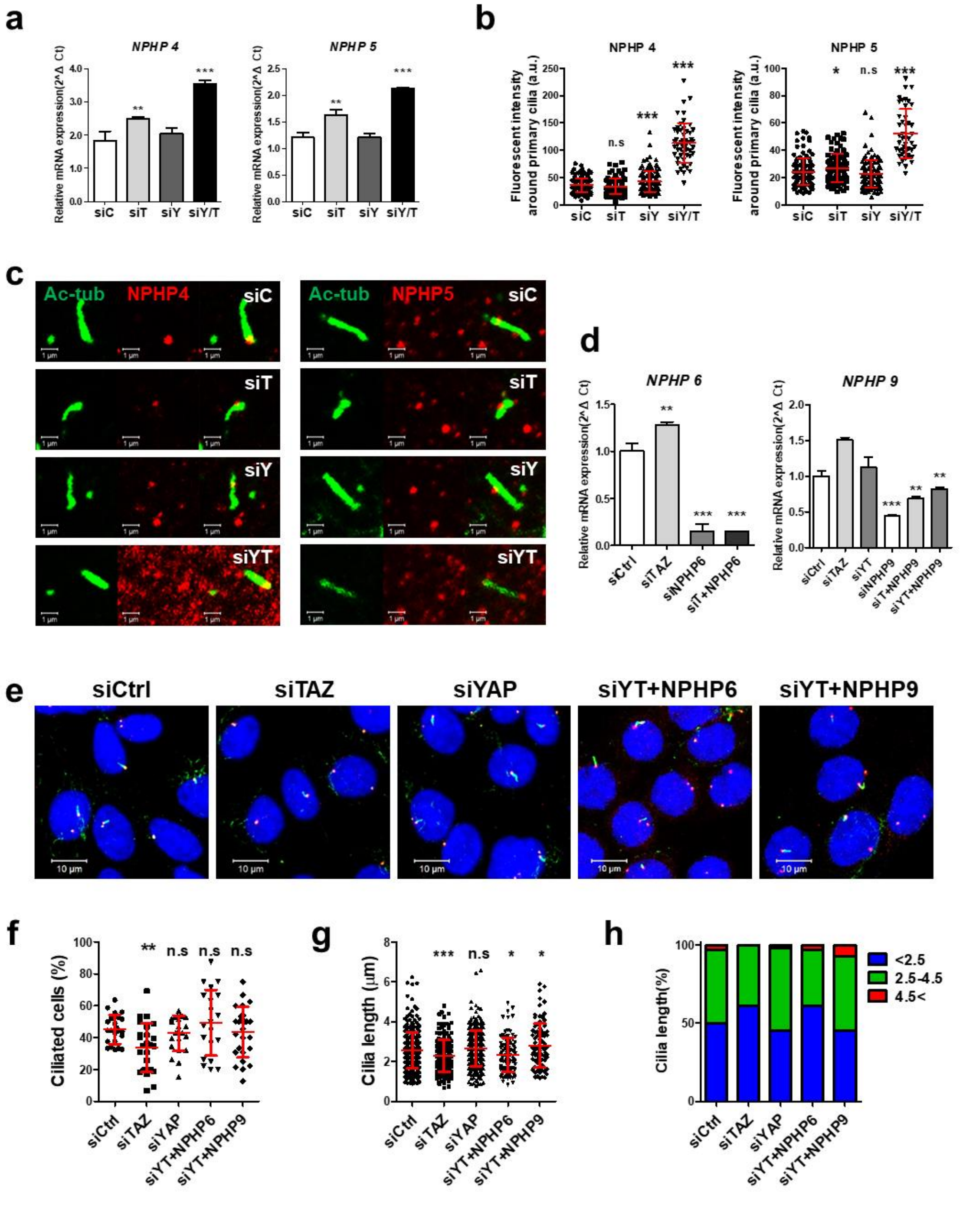

Supplementary Fig 5. NPHP4, 5 with no noticeable difference in single TAZ reduction but ciliary rescue by NPHP6, 9 decrease under YAP/TAZ silencing.

Supplementary Figure Legend

Supplementary Figure Legend

Supplementary Fig 1. Little differences in 2KW/TBW ratio between WT and TAZcKO at early stages and Cre-recombinase expression with KSP-cre mice.

(a) The ratio of 2 kidney weight to total body weight of Taz-floxed and TAZ-floxed:KSP-cre mouse at 3-, 8-, 21-weeks. n.s., not significant. (b-c) Immunofluorescence images of with or without KSP-cre. The renal tubule markers synaptopodin, LTL, calbindin, and DBA were stained together to confirm the expression of Cre-recombinase. (b) No Cre signals were confirmed in WT, but most of the Cre expression was confirmed in the rental tube of cKO. (c) Magnified image with focus on glomerulus and (right bottom) immunohistochemistry stained with Cre-recombinase. White arrows indicate Cre-expressing cells in the capsule region surrounding the glomerulus.

Supplementary Fig 2. YAP and TAZ expression on renal tubule cells under standard condition and during serum starvation.

(a) Western blot analysis to identify the basal level of YAP/TAZ in each cell line. TKPTS; mouse renal cortex proximal tubule cell, mIMCD-3; mouse renal medulla/collecting duct cell, SV40MES13; mouse renal glomerular mesangial cell. (b) Another western blot analysis to identify basal expression level of phosphorylated LATS and TAZ in each renal tubule cell lines. (c) Comparison of YAP/TAZ expression under serum withdrawal at 0 h, 6 h, 12 h, 24 h after FBS removal by immunoblot analysis in mIMCD, SV40MES13 and TKPTS cells.

Supplementary Fig 3. Protein expressions under siRNA transfection.

(a-c) Cell seeding with 4 x 10^4 cell/ml. Subsequent siRNA transfection and after 24 h, change the media with no serum. (a) IMCD (b) SV40MES13 (c) TKPTS cells. Serum-starvation for 24 h or 48 h. Each siRNA was 20 nM-treated and 10 nM-treated with both YAP/TAZ. Immunoblotting to confirm knock-down of YAP/TAZ via siRNA.

Supplementary Fig 4. No significant changes IFT88 expression under YAP and TAZ decrease in SV40MES13.

(a) Immunocytochemistry analysis with IFT88 (red) and cilia, basal body (green) under YAP or TAZ knocked down and 24 hours serum withdrawal in SV40MES13. (b) Each of YAP and TAZ was reduced and the fluorescence intensity compared. Measuring IFT88 intensity of fluorescent with cilia

by ZEN blue software. (c) The localization of IFT88 distribution on cilia was graphed according to the ratio. Both the ciliary tip and the base are located, but if the tip intensity is high, tip>base, if it is the same, tip=base, and if the base intensity is high, it is displayed as tip<base. Also, the case where the positions are evenly distributed on the overall cilia and the case where they are not distributed are described as an even distribution and an irregular distribution, respectively. Mean± SD, ***P<0.001

Supplementary Fig 5. NPHP4, 5 with no noticeable difference in single TAZ reduction but ciliary rescue by NPHP6, 9 decrease under YAP/TAZ silencing.

(a) *NPHP4* and *NPHP5* mRNA expression change under YAP/TAZ knockdown with 24 h serum withdrawal in SV40MES13. (b, c) NPHP4 and NPHP5 were subjected to immunofluorescent staining with primary cilia. (b) Measuring NPHP4 and NPHP5 fluorescence intensity around cilia and basal body were conducted by Zen blue software. Each of YAP and TAZ was silenced and the fluorescence intensity of NPHP 4 and 5 were compared. (c) Images of NPHP4 and NPHP5 expression with primary cilia under declined YAP/TAZ condition. In YAP/TAZ double knocked down condition, endogenous expression of NPHP4, 5 increased with shortening primary cilia, while there was no difference in TAZ knocked down condition. Scaled bar was 1㎛ (d) *NPHP6* and *NPHP9* mRNA expression decreased in each siRNA transfection. (e-h) Primary cilia rescue under YAP and TAZ silencing with NPHP6 or 9 knocked down together in 24 h serum starvation. (e) Fluorescence images showed primary cilia under TAZ or YAP orYAP/TAZ and NPHP 6 and 9 triple knocked down with 24 h serum starvation. Scaled bar was 10㎛ (f) The graphs represented the ratio of ciliated cells to the number of DAPI per images. (g) Primary cilia lengths were measured by ZEN black program. (h) The graphs represented the ratio of cilia lengths within three ranges, less than 2.5 µm, 2.5 µm to 5 µm, and more than 5 µm. Mean± SD, *P<0.05, **P<0.01, ***P<0.001, n.s., not significant
